# Cardiac Mesenchymal Cells Cultured at Physiologic Oxygen Tension Have Superior Therapeutic Efficacy in Heart Failure Caused by Myocardial Infarction

**DOI:** 10.3389/fcell.2021.662415

**Published:** 2021-05-26

**Authors:** Robi A. R. Bolli, Asma Arshia, Syed A. Hassan, Chandrashekhar Dasari, Yibing Nong, Yiru Guo, Alex A. Tomlin, Qianhong Li

**Affiliations:** Department of Medicine, Institute of Molecular Cardiology, University of Louisville, Louisville, KY, United States

**Keywords:** stem cells, heart failure, myocardial infarction, cardiac function, cardiac repair, cardiac mesenchymal cells, physiologic oxygen tension

## Abstract

Stem/progenitor cells are usually cultured at atmospheric O_2_ tension (21%); however, since physiologic O_2_ tension in the heart is ∼5%, using 21% O_2_ may cause oxidative stress and toxicity. Cardiac mesenchymal cells (CMCs), a newly discovered and promising type of progenitor cells, are effective in improving left ventricle (LV) function after myocardial infarction (MI). We have previously shown that, compared with 21% O_2_, culture at 5% O_2_ increases CMC proliferation, telomerase activity, telomere length, and resistance to severe hypoxia *in vitro*. However, it is unknown whether these beneficial effects of 5% O_2_ *in vitro* translate into greater therapeutic efficacy *in vivo* in the treatment of heart failure. Thus, murine CMCs were cultured at 21% or 5% O_2_. Mice with heart failure caused by a 60-min coronary occlusion followed by 30 days of reperfusion received vehicle, 21% or 5% O_2_ CMCs via echocardiography-guided intraventricular injection. After 35 days, the improvement in LV ejection fraction effected by 5% O_2_ CMCs was > 3 times greater than that afforded by 21% O_2_ CMCs (5.2 vs. 1.5 units, *P* < 0.01). Hemodynamic studies (Millar catheter) yielded similar results both for load-dependent (LV dP/dt) and load-independent (end-systolic elastance) indices. Thus, two independent approaches (echo and hemodynamics) demonstrated the therapeutic superiority of 5% O_2_ CMCs. Further, 5% O_2_ CMCs, but not 21% O_2_ CMCs, significantly decreased scar size, increased viable myocardium, reduced LV hypertrophy and dilatation, and limited myocardial fibrosis both in the risk and non-infarcted regions. Taken together, these results show, for the first time, that culturing CMCs at physiologic (5%) O_2_ tension provides superior therapeutic efficacy in promoting cardiac repair *in vivo*. This concept may enhance the therapeutic potential of CMCs. Further, culture at 5% O_2_ enables greater numbers of cells to be produced in a shorter time, thereby reducing costs and effort and limiting cell senescence. Thus, the present study has potentially vast implications for the field of cell therapy.

## Introduction

Cell therapy is emerging as a potentially useful approach to the treatment of heart failure. Cardiac mesenchymal cells (CMCs), a newly discovered and promising type of progenitor cells recently isolated from the heart of mice, rats, and humans, have shown significant efficacy in improving cardiac function in mice and rats that underwent myocardial infarction (MI) resulting in heart failure (Guo et al., 2017; Wysoczynski et al., 2017). In addition to their efficacy, CMCs offer several advantages over other cell types, including the fact that their isolation and expansion are considerably simpler and less expensive than other cardiac-derived cells (Wysoczynski et al., 2017).

Since the establishment of the first cell line culture for mouse fibroblasts in 1943 (Earle et al., 1943), cell culture at normoxic conditions (atmospheric oxygen tension, 21%) has been the standard method used by almost all investigators that use stem or progenitor cells in preclinical and in clinical studies. However, physiologic oxygen tension in living organs is only 3–12% (Mas-Bargues et al., 2019); in the heart, it is ∼5% (Roy et al., 2003). This raises the concern that culturing cells at 21% O_2_ may cause toxicity due to oxidative stress. Furthermore, cells grown at 21% O_2_ may not survive well in the microenvironment of the heart where oxygen tension is 5%, and particularly in the scarred regions where O_2_ is very low. Indeed, many studies in animal models have documented that the vast majority (>95%) of cells transplanted in the heart die or vanish shortly after administration (Wysoczynski et al., 2018). Previous studies of cells other than CMCs suggest that culturing cells at physiological oxygen tension is beneficial (Mohyeldin et al., 2010; Drela et al., 2014; Jagannathan et al., 2016). We have recently demonstrated that the use of physiologic (5%) oxygen tension to culture CMCs *in vitro* improves cell morphology, markedly decreases cell size, markedly increases cell proliferation, and greatly enhances cell resistance to severe hypoxic stress (Bolli et al., 2021). However, it is unknown whether these beneficial effects of 5% oxygen *in vitro* translate into greater therapeutic efficacy *in vivo* in the treatment of heart failure.

The present investigation was undertaken in follow-up to our previous work *in vitro* (Bolli et al., 2021). The goal of this study was to directly compare the therapeutic efficacy of CMCs cultured at physiologic oxygen tension (5%) with that of CMCs cultured with the commonly used atmospheric oxygen tension (21% O_2_) in a mouse model of heart failure caused by an old MI. We found that CMCs cultured at 5% O_2_ had significantly greater therapeutic efficacy; that is, they produced a greater improvement in left ventricular (LV) function, a greater increase in viable myocardium, and a greater decrease in myocardial fibrosis.

## Materials and Methods

### Ethics Statement

All animal procedures were performed in accordance with the Guide for the Care and Use of Laboratory Animals (Department of Health and Human Services, Publication No. [NIH] 86–23) and with the guidelines of the Animal Care and Use Committee of the University of Louisville, School of Medicine (Louisville, KY, United States). The protocol was approved by the Institutional Animal Care and Use Committee of the University of Louisville. All the surgical procedures, measurements of LV function (echocardiography and hemodynamic studies) and pathological analyses were performed by investigators double-blinded to treatment allocation.

### Isolation of Murine CMCs

Male C57BL/6J mice (The Jackson Laboratory), 12 ± 0.5 weeks old, were euthanized by sodium pentobarbital injection (100 mg/kg, i.p.). The hearts were excised, rinsed with PBS (pH 7.4) at room temperature, minced into small pieces, and then enzymatically dissociated with Collagenase II (5 μg/mL in PBS, ≥ 125 units per mg dry weight; Worthington) via gentle agitation at 37°C for 45 min. After Collagenase II inactivation with DMEM/F12 medium containing 10% FBS, cells were centrifuged at 600 g for 10 min at room temperature. The collected cell pellet was suspended in growth medium consisting of DMEM/F12 (Invitrogen), 10% FBS (Seradigm, VWR), bFGF (10 ng/ml, Invitrogen), EGF (10 ng/ml, Invitrogen), ITS (insulin/transferrin/selenium, Invitrogen), glutamine (Invitrogen), and penicillin streptomycin solution (Invitrogen). A single-cell suspension was plated into culture plates. The non-adherent cells were removed 2 h after initial plating, and the attached cells were gently rinsed with PBS and then continuously cultured in new growth medium. Collected floating cells were plated into new cell culture plates and cultured for the next 2 h. The procedure was repeated three more times at 24, 48, and 72 h, respectively. A total of five fractions were collected after myocardial digestion. The cell fractions that attached within the first 2 and 4 h were discarded, as most of them were cardiac fibroblasts, which can quickly attach on the culture plates, whereas the cells that attached on the culture plates at 24, 48, and 72 h were designated as cardiac mesenchymal cells (CMCs), whose properties and function have been identified previously (Wysoczynski et al., 2017).

### Murine CMC Culture

Murine CMCs were cultured in growth medium (Wysoczynski et al., 2017; Schulman et al., 2018). Under a microscope, when cells reached ∼80% confluence in the culture plate, cell images were acquired to monitor changes in cell morphology. Then, cells from one 100-mm culture plate were digested by trypsinization, counted with a hemocytometer under a microscope, split and seeded into new 100-mm culture plates at 5,000 cells/cm^2^. Beginning at passage 2, cells were cultured either in a 21% O_2_ or 5% O_2_ incubator supplied with 5% CO_2_ at 37°C. Cells at passages 5–6 were employed for cell transplantation *in vivo*. To control the quality of the cell batch, the same batch of cells at passage 2 characterized previously (Wysoczynski et al., 2017) was used to develop 21% and 5% O_2_ CMCs for the *in vitro* studies of cell competence and functional properties (Bolli et al., 2021). The same batch of these 21% and 5% O_2_ CMCs used in those *in vitro* studies was employed for the current study *in vivo*. In all experiments, CMCs were passaged <7 times (Bolli et al., 2021).

### Murine Model of Heart Failure Caused by an Old MI

**FIGURE 1:**
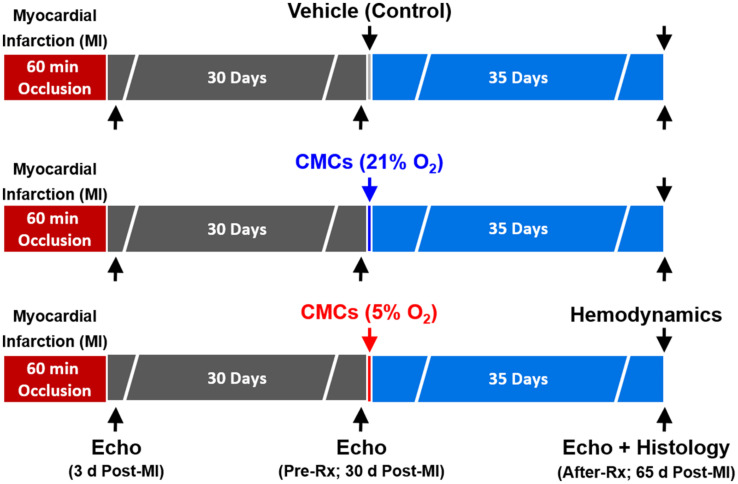
Experimental protocol. Mice were subjected a 60-min coronary occlusion (myocardial infarction [MI]) followed by 65 days of reperfusion. Thirty days after MI, mice received vehicle, or 21% or 5% O_2_ cultured CMCs (1 × 10^6^ cells) via echocardiography-guided intraventricular infusion. Serial echocardiographic studies were performed at 3 days post-MI, 30 days post-MI (before treatment) (Pre-Rx) and 35 days after treatment (After-Rx, 65 days post-MI); *n* = 16/group. At the conclusion of the protocol, mice underwent a hemodynamic study and were euthanized, after which the hearts were harvested for morphometric and histopathologic analyses.

C57BL/6J female mice (weight, 22.4 ± 0.4 g; age 14–15 weeks) purchased from The Jackson Laboratory (Bar Harbor, ME, United States) were used. Mice were maintained in microisolator cages under specific pathogen-free conditions in a room with a temperature of 24°C, 55–65% relative humidity, and a 12-h light–dark cycle. Mice were anesthetized with sodium pentobarbital (60 mg/kg i.p.) and ventilated using carefully selected parameters. The chest was opened through a midline sternotomy, and a non-traumatic balloon occluder was implanted around the mid-left anterior descending coronary artery using an 8-0 nylon suture. To prevent hypotension, blood from a donor mouse was given at serial times during surgery. Rectal temperature was carefully monitored and maintained between 36.7 and 37.3°C throughout the experiment. In all groups, MI was produced by a 60-min coronary occlusion followed by reperfusion (Figure 1). Successful performance of coronary occlusion and reperfusion was verified by visual inspection (i.e., by noting the development of a pale color in the distal myocardium after inflation of the balloon and the return of a bright red color due to hyperemia after deflation) and by observing ST-segment elevation and widening of the QRS on the ECG during ischemia and their resolution after reperfusion). Mice were then followed for 65 days (Guo et al., 2017; Wysoczynski et al., 2017; Guo et al., 2020).

### Echocardiography-Guided Intraventricular CMC Transplantation

Thirty days after MI, mice were randomly allocated to vehicle or different cell-treated groups. CMC or vehicle transplantation was performed using a Vevo 2100 Imaging System (VisualSonics, Inc.) equipped with a 30-MHz transducer, a Vevo Image Station with Injection Mount, and micro-manipulation controls (Guo et al., 2017; Nong et al., 2020). Mice were reanesthetized with isoflurane (3% for induction, 1.5% for maintenance). The anterior chest was shaved and mice were placed on the imaging table in the right lateral decubitus position with the left lateral side facing the injection mount. After a good long-axis view of left ventricle was procured, the transducer was turned 90 degrees clockwise. The left ventricle was scanned in the short-axis view from apex to base to determine the optimal site for needle insertion. To prevent bleeding from the LV wall, it is crucial to find a site that avoids the infarct scar and coronary arteries. Under real-time B-mode view, a 0.5-inch 30 G needle connected to a 1.0 ml syringe was carefully inserted from the left lateral side of the chest and advanced into the center of the LV cavity. Successful penetration of the LV cavity was indicated by a small reflux of bright red blood from the needle into the syringe tip. CMCs (1 × 10^6^ cells in 200 μl of PBS) or vehicle (200 μl of PBS) were infused at a steady rate of 2.2 μl/s over 90 s. After the infusion, the needle was quickly withdrawn from the left ventricle. Using a rectal temperature probe, body temperature was controlled in the range of 37 ± 0.2°C, and the electrocardiogram and respiration were monitored carefully during the whole procedure. Mice were allowed to recover in a temperature-controlled area (Guo et al., 2017).

### Echocardiographic Studies

The echocardiographic studies were performed using the Vevo 2100 Imaging System (VisualSonics, Inc.) equipped with a 30-MHz transducer, as previously described (Li Q. et al., 2011). Serial echocardiograms were obtained at 3 days after the 60-min occlusion (Post-MI), 30 days after MI (before treatment, Pre-Rx), and 35 days after treatment (After-Rx) (Figure 1) under isoflurane anesthesia (3% for induction, 1% for maintenance). Using a rectal temperature probe, body temperature was carefully maintained at 37 ± 0.2°C throughout the study. The parasternal long axis and parasternal short-axis views were used to obtain 2D mode images for the measurement of LV mass, end-diastolic and end-systolic LV volume (LVEDV and LVESV), stroke volume (SV), and EF, as previously described (Dawn et al., 2006; Li Q. et al., 2011). Digital images were analyzed off-line by blinded observers using the Vevo 2100 software. Measurements were performed according to the American Society for Echocardiography (Gottdiener et al., 2004; Mehra et al., 2018). At least three measurements were acquired and averaged for each parameter (Gao et al., 2011).

### Hemodynamic Studies

To avoid any potential effects of isoflurane anesthesia on cardiac function, hemodynamic studies were performed 4 days after the final echocardiography studies and just before euthanasia, as previously reported (Guo et al., 2017; Mehra et al., 2018). Mice were anesthetized with sodium pentobarbital (60 mg/kg i.p.), intubated, and ventilated with a positive pressure ventilator (Hugo-Sachs Electronik D-79232 [Germany]; ventilation rate, 105/min; tidal volume 10.3 μl/g). Rectal temperature was kept at 37 ± 0.2°C. A 1.0 French pressure–volume (PV) catheter (PVR-1035, Millar Instruments) was inserted into the left ventricle via the right carotid artery. The position of the catheter was carefully adjusted until typical and stable PV loop signals were acquired. After 30 min of stabilization, the PV signals were recorded continuously with an MPVS ULTRA Pressure-Volume Unit (Millar Instruments) coupled with a Powerlab 16/30 converter (AD Instruments), stored, and displayed on a computer with LabChart 7.0 software (AD Instruments). Inferior vena cava occlusions were performed with external compression to produce variably loaded beats for determination of the end-systolic PV relation and other derived constructs of LV performance. Parallel conductance from surrounding structures was calculated by a bolus injection of 5 μl of 30% NaCl through the jugular vein. Echocardiography-derived SV was used as outside reference for alpha calibration for LV volume. All hemodynamic data analyses were performed off-line using LabChart 7.0 software by an investigator blinded to the treatment allocation (Li Q. et al., 2011).

### Histological Studies

At the conclusion of the protocol, the heart was arrested in diastole by an i.v. injection of 0.15 ml of CdCl_2_ (100 mM), excised, and perfused retrogradely at 60–80 mmHg (LVEDP = 8 mmHg) with heparinized PBS for 2 min, followed by 10% neutral buffered formalin solution for 15 min. The heart was then sectioned into three slices from apex to base, fixed in formalin for 24 h, and subjected to tissue processing and paraffin embedding. Paraffin-embedded LV blocks were sectioned at a thickness of 4 μm for histological studies (Li Q. et al., 2011). Morphometric parameters, including LV cavity area, total LV area, risk region area, scar area, LV wall thickness, and infarct expansion index were measured in sections stained with Masson’s trichrome as described previously (Tang et al., 2016). Myocardial collagen content was quantitated on picrosirius red-stained heart images acquired with polarized light microscopy by determining collagen area per mm^2^ of risk region or non-infarcted region (Tang et al., 2018). All acquired images were analyzed using NIH ImageJ software (version 1.52a) and measurements were averaged from three slides (trichrome) or two slides (collagen) per heart (Guo et al., 2017; Tang et al., 2018).

### Statistical Analysis

The statistical methods were similar to those previously used by our group (Bennett et al., 1987; Triana et al., 1991; Li et al., 1993, 2001; Tang et al., 1996; Dawn et al., 2008). Data are presented as mean ± SEM. Data were analyzed with one*-*way repeated measures ANOVA as well as with one-way ANOVA for normally distributed data, or Kruskal–Wallis one-way analysis of variance on ranks for data that are not normally distributed, as appropriate, followed by unpaired Student’s *t*-tests with the Bonferroni correction. A *P* < 0.05 was considered statistically significant. All statistical analyses were performed using the SigmaStat software system (3.5 V).

## Results

### Exclusions

A total of 96 mice were used for this study. Forty-one mice died during or shortly after the surgical procedure of opening the chest and producing a 60-min coronary occlusion to induce MI. Seven mice were excluded because of technical problems, including body temperature out of normal range (*n* = 1), balloon occluder malfunction (*n* = 1), bleeding after echocardiography-guided LV cell injection (*n* = 2), and poor echocardiographic images for data analysis (*n* = 3). Thus, a total of 48 mice were included in the final analysis.

### Fundamental Physiological Parameters

During the echocardiographic studies, heart rate and body temperature (fundamental physiological parameters that may impact myocardial function) were similar in the three groups. The heart rate was 516 ± 10 beats/min in the vehicle group, 520 ± 11 beats/min in the 21% O_2_ cultured CMC group, and 495 ± 7 beats/min in the 5% O_2_ cultured CMC group. By experimental design, rectal temperature remained within a narrow, physiologic range (36.9–37.1°C) in all three groups (Table 1).

**TABLE 1:** Rectal temperature and heart rate on the days of echocardiography assays.

	Post MI	Pre Rx	After Rx

	(3 days after MI)	(30 days after MI)	(35 days after Rx)
**Temperature (°C)**			
Vehicle Group	36.9 ± 0.1	37.1 ± 0.1	36.9 ± 0.0
21% O_2_ CMC Group	36.9 ± 0.1	36.9 ± 0.0	37.0 ± 0.0
5% O_2_ CMC Group	37.0 ± 0.1	37.0 ± 0.0	37.0 ± 0.0
**Heart rate (beats/min)**			
Vehicle Group	516 ± 10	487 ± 9	483 ± 9
21% O_2_ CMC Group	520 ± 11	499 ± 8	511 ± 7
5% O_2_ CMC Group	495 ± 7	482 ± 9	495 ± 10

**Measurements of rectal temperature and heart rate were acquired during the echocardiographic studies (n = 16/group). Data are means ± SEM.**

### Effect of Physiologic Oxygen Tension on CMCs

**FIGURE 2:**
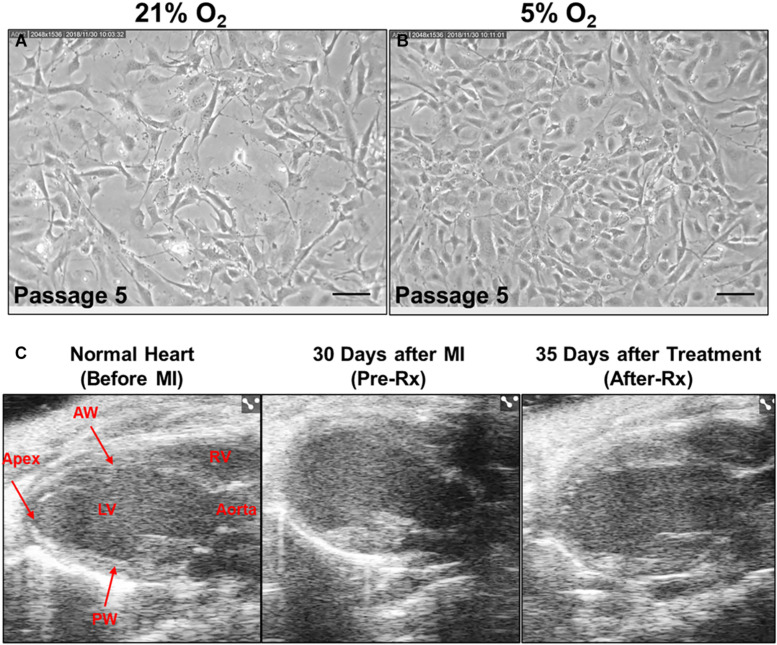
Effect of CMCs cultured at 5% oxygen tension on cardiac function after MI. Representative microscopic images of CMCs acquired from 21% **(A)** and 5% **(B)** O_2_ cultured CMCs at passage five under magnification × 200. Scale bars, 100 μm. **(C)** Representative echocardiography B-mode long-axis images obtained before MI (*left*), 30 days after MI (Pre-Rx) (*middle*) and 35 days after 5% O_2_ cultured CMC treatment (After-Rx) (*right*). LV, left ventricle; RV, right ventricle; AW, anterior wall; PW, posterior wall.

After they were isolated from the mouse heart, CMCs were cultured in an incubator supplied either with 21% or 5% O_2_ beginning at passage 2. As illustrated in Figure 2A, CMCs cultured at 21% O_2_ for 4 days at passage 5 showed poor morphology and exhibited lower cell density; cells were large and elongated with varied shapes. In contrast, CMCs cultured at 5% O_2_ for 4 days at passage 5 were small, showed higher density, and had round/oval and more uniform shape (Figure 2B). These observations are consistent with our previous studies (Bolli et al., 2021) and suggest that physiological 5% O_2_ is beneficial to CMC function.

### Effect of CMCs Cultured at Physiologic Oxygen Tension on LV Function Measured by Echocardiography

**FIGURE 3:**
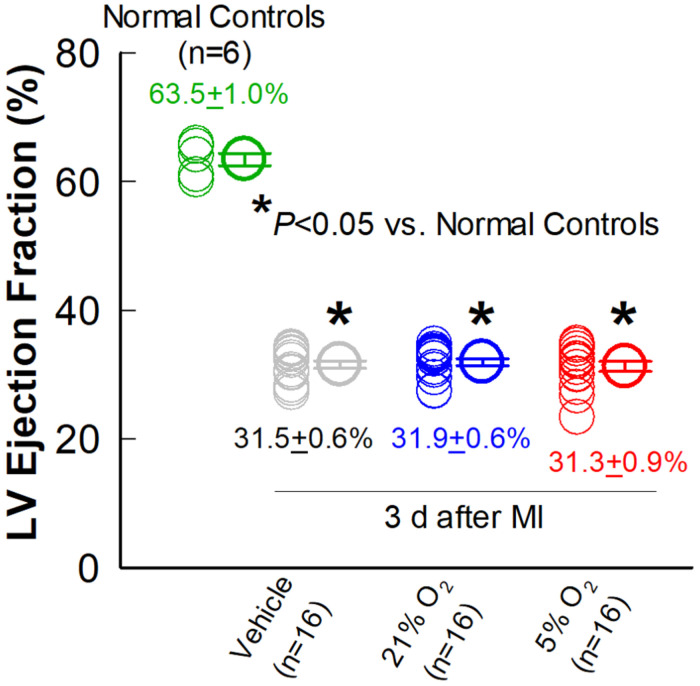
Comparison of LV EF three days after MI. Echocardiographic measurements of LV EF were performed in the vehicle, 21% O_2_, and 5% O_2_ CMC groups (*n* = 16/group) 3 days after MI (in the absence of cell treatment) and in normal, age-matched control mice that did not undergo surgery (*n* = 6). Data are means ± SEM. *P < 0.05 vs. normal control group.

Echocardiographic studies were performed 3 days after MI (Post-MI), 30 days after MI (before treatment) (Pre-Rx), and 35 days after treatment (After-Rx, 65 days after MI). According to our inclusion criteria, only mice with severe LV dysfunction, i.e., EF < 35% at 30 days after MI, were included in the study (Figure 3). Representative echocardiographic images acquired from a mouse in the 5% O_2_ CMC group before MI, 30 days after MI (Pre-Rx), and 35 days after cell treatment (After-Rx), are presented in Figure 2C.

**FIGURE 4:**
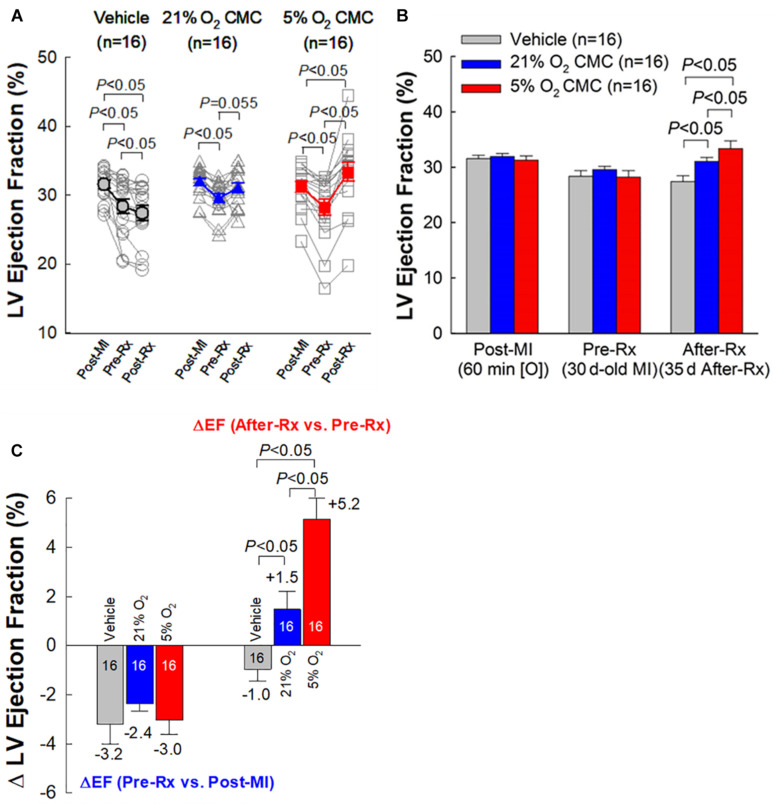
Echocardiographic assessment of LV EF. **(A)** Values of LV EF acquired from each individual mouse at each time-point. **(B)** Mean values of LV EF at each time-point. **(C)** Changes in LV EF. On the *left* are depicted the changes in LV EF (absolute units) between 30 days after MI (before treatment) (Pre-Rx) and 3 days after MI (Post-MI). On the *right* are depicted the changes in LV EF (absolute units) between 35 days after treatment (After-Rx) and 30 days after MI (before treatment) (Pre-Rx). *n* = 16/group. Data are mean ± SEM.

As expected, the vehicle group showed a significant, progressive deterioration in LV function, with the average EF decreasing from 31.5 to 28.4% (*P* < 0.05) in the 30-day interval between 3 days post-MI and right before vehicle injection (Pre-Rx); in this group, LV EF continued to decline from 28.4 to 27.4% (*P* < 0.05) in the subsequent 35-day interval after vehicle injection (After-Rx) (Figure 4A).

**FIGURE 5:**
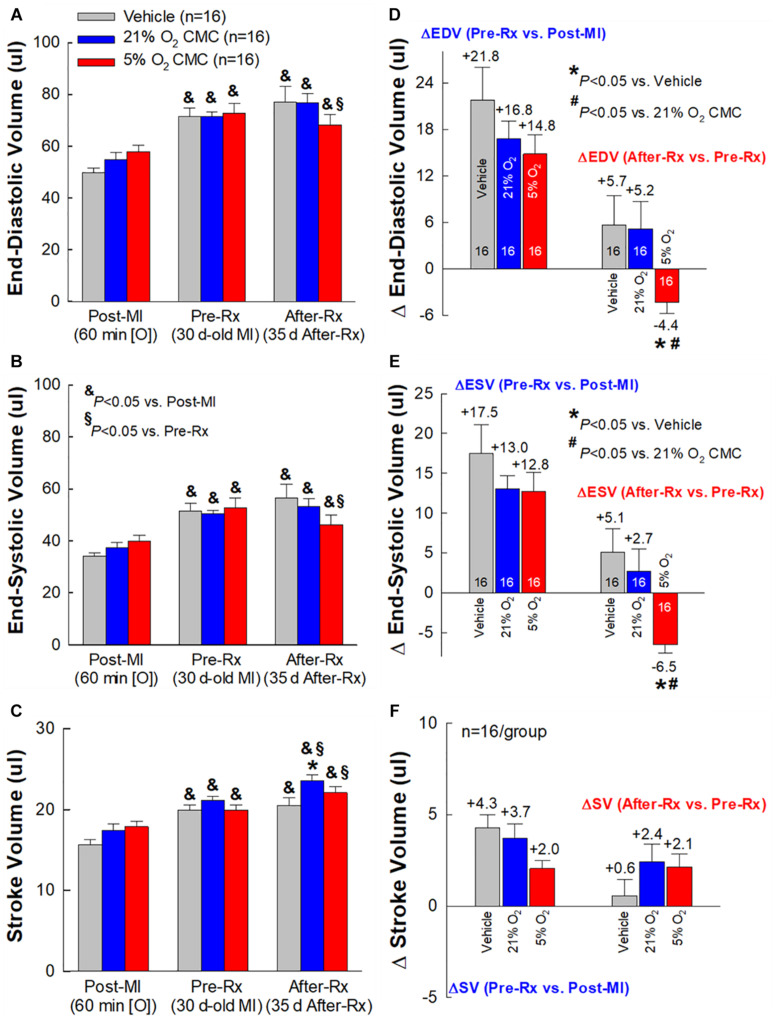
Echocardiographic assessment of LV volumes. Serial echocardiographic measurements of LV end-diastolic volume (EDV) **(A)**, LV end-systolic volume (ESV) **(B)** and LV stroke volume (SV) **(C)**, were performed 3 days after a 60-min coronary occlusion/reperfusion (Post-MI), 30 days post-MI (before treatment) (Pre-Rx), and 35 days after treatment (After-Rx, 65 days post-MI). The right panels depict the changes in LV EDV **(D)**, ESV **(E),** and SV **(F)** between 30 days post-MI (before treatment) (Pre-Rx) and 3 days after MI (Post-MI) (*left*), and between 35 days after treatment (After-Rx) and 30 days post-MI (before treatment) (Pre-Rx) (*right*). *n* = 16/group. Data are means ± SEM. **^&^***P* < 0.05 vs. the same group Post-MI; **^§^** *P* < 0.05 vs. the same group Pre-Rx; *P < 0.05 vs. vehicle group at the same time-point; **^#^***P* < 0.05 vs. 21% O_2_ CMC group at the same time-point.

At 30 days post-MI (before treatment) (Pre-Rx), there were no significant differences among the three groups with respect to LV end-diastolic volume (LVEDV), end-systolic volume (LVESV), stroke volume (SV), and EF (Figures 4A,B, 5A–C), indicating that the severity of post-MI LV remodeling and dysfunction was comparable in all groups. The pronounced LV dilatation in all groups, as shown by the increase in LVEDV and LVESV (Figures 5D,E), demonstrates that this murine model exhibits significant LV remodeling after MI. Thirty-five days after treatment (After-Rx), a significant improvement in LV EF was noted both in the 21% and 5% O_2_ CMC groups compared with that in the vehicle group; however, the improvement was significantly more robust in the 5% O_2_ CMC group. This is demonstrated by the fact that in this group, the increase in LV EF from pretreatment values (Pre-Rx) to 35 days after treatment (After-Rx) was 5.2 ± 0.9% (absolute units), whereas in the 21% O_2_ CMC group it was only 1.5 ± 0.7% (*P* < 0.05 vs. the 5% O_2_ CMC group) (in the vehicle group, LV EF actually decreased by –1.0 ± 0.5%) (Figure 4C). As a result, at 35 days after treatment LV EF was significantly (*P* < 0.05) greater in the 5% O_2_ CMC group compared with the 21% O_2_ CMC group (Figures 4A,B). A similar pattern was also observed in ΔLVEDV, ΔLVESV and ΔSV: both 21% O_2_ CMCs and 5% O_2_ CMCs improved LVEDV, LVESV and SV compared with vehicle (Figures 5D–F), but the improvement in the 5% O_2_ CMC group was statistically greater than that in the 21% O_2_ CMC group (Figures 5D,E). One*-*way repeated measures ANOVA showed that among the three groups, only 5% O_2_ CMCs significantly improved LV EF (Figure 4A) and LV remodeling (EDV and ESV; Figures 5A,B) at 35 days after treatment as compared with pre-treatment values.

Taken together, these results indicate that 5% O_2_ CMCs are superior to 21% O_2_ CMCs in improving the function of the failing murine heart after a 30-day old MI and that the use of physiologic oxygen tension (5%) to culture CMCs increases their therapeutic efficacy.

### Effect of CMCs Cultured at Physiologic Oxygen Tension on LV Function Measured by Hemodynamic Studies

**FIGURE 6:**
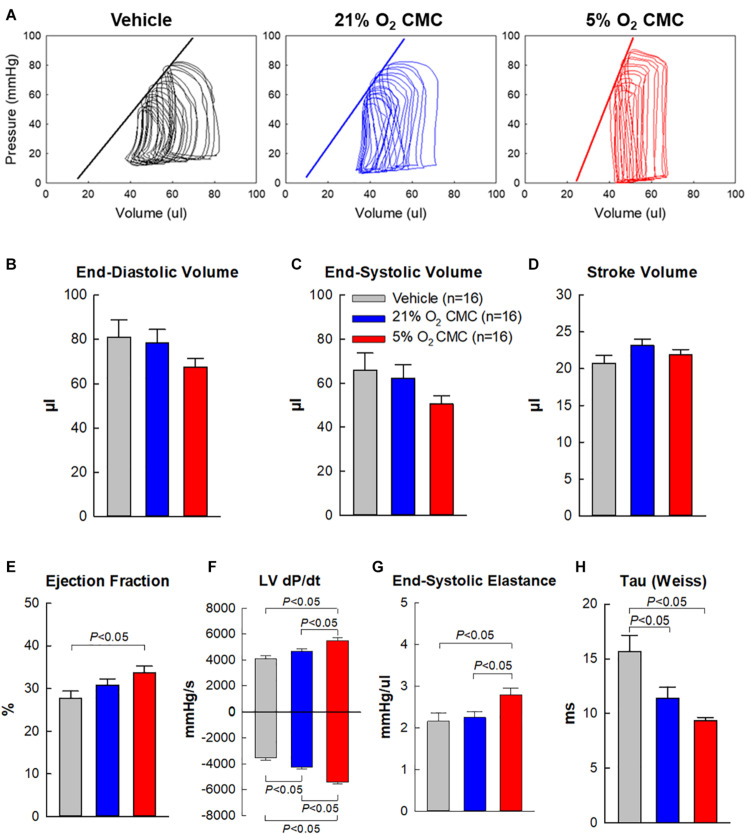
Hemodynamic assessment of cardiac function. Hemodynamic studies were performed with a 1F Millar conductance catheter 39 days after treatment (4 days after the final echocardiographic study), just before euthanasia. **(A)** Representative LV pressure-volume loops recorded during preload manipulation by brief inferior vena cava occlusions. **(B–H)** Quantitative analysis of hemodynamic variables. Data are means ± SEM.

To prevent any after-effects of the anesthesia used during the echocardiographic assessment 35 days after cell administration, a 4-day interval was allowed between echocardiographic and hemodynamic measurements; thus, the hemodynamic studies were performed just before euthanasia, 39 days after vehicle or cell administration. At this time, all LV functional parameters were markedly depressed in the vehicle, 21% and 5% O_2_ CMC groups (Figure 6). Figure 6A illustrates representative LV pressure-volume (P-V) loops acquired in the vehicle, 21% and 5% O_2_ CMC groups. The leftward shift of the volume signal and increased amplitude of the pressure signal in the P-V loops (resulting in a steeper end-systolic pressure-volume relationship) in the 5% O_2_ CMC-treated mouse are indicative of reduced LV operating volumes and enhanced contractility. Compared with the vehicle group, hemodynamic parameters were improved in both cell-treated groups, but the improvement was more robust in the 5% O_2_ CMC group than in the 21% O_2_ CMC group. This was the case not only for load-dependent (LV EF and LV dP/dt) but also for load-independent (end-systolic elastance and Tau) indices of LV function (Figures 6E–H). Both LV dP/dt and end-systolic elastance were significantly greater in the 5% O_2_ CMC group than in the 21% O_2_ CMC group, and EF was improved in the former but not in the latter (Figures 6E–G). Thus, two independent methods of functional assessment (echocardiography and hemodynamic studies) consistently demonstrated that the functional benefits offered by 5% O_2_ CMC treatment are superior to those of 21% O_2_ CMC treatment.

### Effect of CMCs Cultured at Physiologic Oxygen Tension on LV Structure

A hallmark of ischemic cardiomyopathy is the presence of compensatory hypertrophy in the surviving myocardium. As shown in Table 2, LV weight was significantly increased in vehicle-treated mice compared with normal controls, indicating the development of compensatory LV hypertrophy after MI in this murine model of chronic ischemic cardiomyopathy. Importantly, the ratios of LV weight to body weight and LV weight to tibial length were significantly reduced in the 5% O_2_ CMC group (*n* = 12, *P* < 0.05) as compared with the vehicle group (*n* = 12, Table 2), demonstrating that administration of 5% O_2_ grown CMCs ameliorated LV hypertrophy. In contrast, these parameters did not differ significantly between the 21% O_2_ and vehicle groups. Although the differences among groups were not statistically significant, both the ratios of lung weight to body weight and lung weight to tibial length were nominally lower in the 5% O_2_ CMC group (but not in the 21% O_2_ CMC group) compared with the vehicle group, suggesting that 5% O_2_ grown CMCs may alleviate pulmonary congestion.

**TABLE 2:** LV hypertrophy parameters.

	**Normal control (*n* = 5)**	**Vehicle (*n* = 12)**	**21% O_2_ CMCs (*n* = 11)**	**5% O_2_ CMCs (*n* = 12)**
LV weight (mg)	109.4 ± 7.5*	126.0 ± 3.0	115.8 ± 3.1*	112.2 ± 3.3*
Body weight (g)	24.8 ± 0.9	23.6 ± 0.2	23.1 ± 0.4	24.3 ± 0.4
LV weight/Body weight (mg/g)	4.5 ± 0.4*	5.4 ± 0.1	5.0 ± 0.2	4.6 ± 0.1*
LV weight/Tibia length (mg/mm)	5.9 ± 0.4*	7.0 ± 0.2	6.4 ± 0.2	6.2 ± 0.2*
Lung weight/Body weight (mg/g)	6.8 ± 0.3	7.4 ± 0.2	7.3 ± 0.2	7.1 ± 0.1
Lung weight/Tibia length (mg/mm)	9.1 ± 0.6	9.8 ± 0.2	9.6 ± 0.2	9.3 ± 0.2

*P < 0.05 vs. vehicle group. Data are means ± SEM.

**FIGURE 7:**
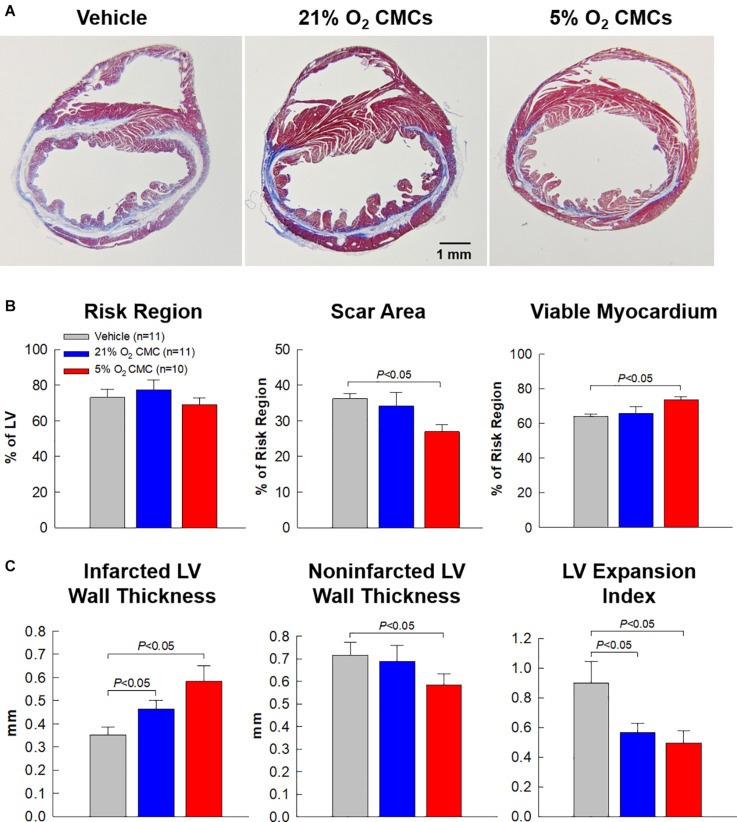
Morphometric analysis of LV remodeling. **(A)** Representative Masson’s trichrome-stained myocardial sections from vehicle, 21% O_2_ CMC, or 5% O_2_ CMC-treated hearts. Scar tissue and viable myocardium are identified in blue and red, respectively. **(B,C)** Quantitative analysis of LV morphometric parameters. Data are mean ± SEM.

The effects of CMC transplantation on LV remodeling were further assessed by Masson’s trichrome staining. Figure 7 shows representative Masson’s trichrome-stained myocardial sections from the vehicle, 21% and 5% O_2_ CMC groups. Scar tissue and viable myocardium are identified in blue and red, respectively. There were no appreciable differences among the three groups with respect to the size of the region at risk (Figure 7B), suggesting that the magnitude of the ischemic insult was comparable in all groups. This is confirmed by the fact that LV EF was similar in all groups after MI, as shown in Figure 4B. Compared with the vehicle group, the 5% O_2_ CMC-treated hearts exhibited a decrease in scar size and a concomitant increase in the amount of viable myocardium in the region at risk (Figure 7B), concomitant with an increase in LV anterior wall (infarct wall) thickness, a decrease in LV posterior wall (non-infarcted wall) thickness, and a decrease in LV expansion index (which is indicative of reduced LV dilatation) (Figure 7C). In contrast, the 21% O_2_ CMC-treated hearts did not differ significantly from the vehicle-treated hearts with respect to scar size, viable myocardium, or non-infarcted wall thickness (Figures 7B,C). The increased amount of viable myocardium may have contributed to the increased cardiac function in mice that received 5% O_2_ CMCs.

### Effect of CMCs Cultured at Physiologic Oxygen Tension on Myocardial Collagen Content

**FIGURE 8:**
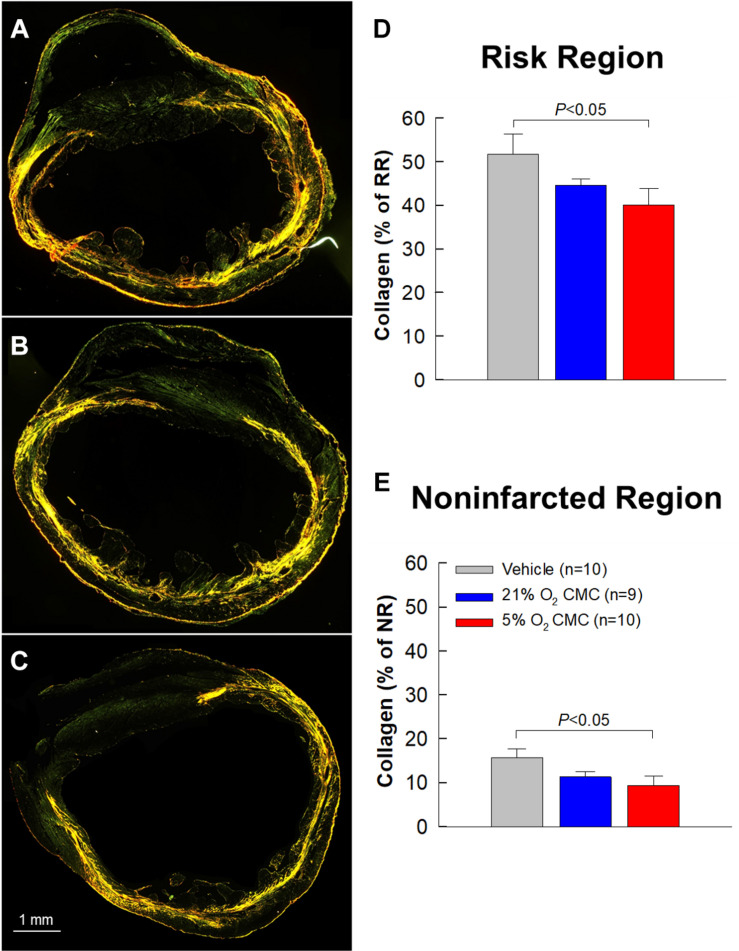
Collagen content in myocardium. **(A–C)** Representative images of LV sections stained with picrosirius red and analyzed with polarized light microscopy from vehicle, 21% O_2_ CMC, or 5% O_2_ CMC-treated hearts. **(D,E)** Quantitative analysis of collagen content in myocardium expressed as a percentage of the risk **(D)** or non-infarcted **(E)** region. Data are mean ± SEM.

One of the features of the failing heart is fibrosis, i.e., excess deposition of collagen in the extracellular matrix. We performed quantitative analysis of collagen content in the heart using picrosirius red staining followed by imaging with polarized light microscopy (Figures 8A–C). Collagen content was evaluated based on stained pixel density using the NIH ImageJ software and expressed as a percentage of the risk or non-infarcted region. In the risk region (which is the sum of the infarcted and border regions), collagen content tended to be less in the 21% O_2_ CMC group compared with the vehicle group but the difference was not statistically significant (Figure 8D). In contrast, collagen content in the risk region was significantly reduced in hearts treated with 5% O_2_ grown CMCs as compared with the vehicle group (40.1 ± 3.8% of risk region vs. 51.6 ± 4.7%, respectively, *P* < 0.05; Figure 8D). Similarly, in the non-infarcted region, collagen content was significantly less in the 5% O_2_ CMC-treated group (9.4 ± 2.1% of non-infarcted region) as compared with the vehicle group (15.7 ± 1.9%, *P* < 0.05; Figure 8E)—a relative reduction of nearly 50%. In contrast, collagen content was not statistically significant different in hearts given 21% O_2_ grown CMCs compared with vehicle, although it tended to be less in the former (Figure 8E). This reduced collagen deposition in the myocardial interstitial space of both risk and non-infarcted regions may have contributed, at least in part, to the functional benefits imparted by 5% O_2_ CMC therapy.

## Discussion

The goal of this study was to compare the therapeutic efficacy of CMCs cultured at atmospheric oxygen tension (21%) (which is the standard method used by almost all investigators that study stem or progenitor cells) with that of CMCs cultured at physiologic oxygen tension (5%). We used a well-established murine model of chronic ischemic cardiomyopathy in which LV dysfunction is produced by a 30-day old MI (Guo et al., 2017; Nong et al., 2020). This model, characterized by severe and progressive LV dysfunction, LV hypertrophy, and myocardial fibrosis, mimics the common clinical setting of chronic HF caused by old MIs, which accounts for approximately half of all cases of HF (Jaarsma and Stromberg, 2000; Amirrasouli and Shamsara, 2017; Benjamin et al., 2019).

The salient findings of this study can be summarized as follows. In mice with LV dysfunction caused by a 30-day old MI, both 21% and 5% O_2_ CMCs improved LV function; however, the improvement was significantly greater with 5% O_2_ CMCs than 21% O_2_ CMCs. For example, relative to control mice, the increase in LV EF was approximately triple in mice treated with 5% O_2_ CMCs compared with mice treated with 21% O_2_ CMCs (5.2 ± 0.9 vs. 1.5 ± 0.7 absolute EF units, respectively; Figure 4). The evidence we provide for the superior therapeutic efficacy of 5% O_2_ CMCs is robust, because it is based on two independent methods to measure LV function (echocardiography and hemodynamic studies) and on load-dependent as well as independent parameters, both of which point consistently to a greater improvement in hearts treated with 5% O_2_ CMCs (Figures 4–6). This functional improvement afforded by 5% O_2_ CMCs was associated with a reversal or prevention of compensatory LV hypertrophy, such that the LV weight/body weight and LV weight/tibia length ratios were similar to those of normal mice (Table 2). The reduction in compensatory LV hypertrophy was likely secondary to improved LV function. The functional improvement in the 5% O_2_ CMC group was also associated with improved LV structure, as shown by thicker infarct scars, thinner non-infarcted LV walls, and markedly reduced LV expansion index, which likely reflected more viable tissue, less compensatory hypertrophy, and less LV dilatation (Figure 7C). Unlike heart treated with 21% O_2_ CMCs, hearts treated with 5% O_2_ CMCs exhibited smaller scars and more viable myocardium (Figure 7). Since we have previously demonstrated that CMCs do not engraft in the heart (Guo et al., 2017; Wysoczynski et al., 2017), these results suggest that administration of 5% O_2_ CMCs may have reduced myocyte apoptosis and/or promoted endogenous myocyte proliferation. Finally, administration of 5% O_2_ CMCs was associated with less fibrosis in the non-infarcted region (Figure 8E), suggesting that these cells were more effective at limiting extracellular matrix remodeling. Taken together, these results demonstrate that 5% O_2_ CMCs are therapeutically superior to 21% O_2_ CMCs in the setting of chronic ischemic cardiomyopathy. Previous studies have shown increased efficacy of other cell types cultured at physiologic oxygen tension (Li T. S. et al., 2011; Amirrasouli and Shamsara, 2017); however, to our knowledge, this is the first report that the therapeutic efficacy of CMCs *in vivo* is greatly enhanced by culturing them at physiologic oxygen tension (5%).

Some methodological aspects of this study deserve comment. Mice underwent a 60-min coronary occlusion followed by 30 days of reperfusion; this model of reperfused MI is more clinically relevant than models of permanent occlusion because in contemporary practice almost all patients with MI undergo spontaneous or iatrogenic reperfusion (Benjamin et al., 2019). Reperfusion dramatically alters the evolution and pathophysiology of MI (Basso and Thiene, 2006). The long (60-min) coronary occlusion produces large infarcts, sufficient to cause heart failure. This is confirmed by the severe, progressive decline in LV function (Figures 4, 5) and the development of structural abnormalities characteristic of the failing heart, i.e., LV hypertrophy (Table 2) and myocardial fibrosis (Figure 8). We allowed mice to recover for 30 days in order to ensure that the infarct has healed completely and a stable scar has formed. This situation is analogous to that of patients with coronary artery disease who have scars caused by old MIs and develop heart failure as a consequence of the loss of cardiac muscle. At 30 days after MI, vehicle or CMCs were delivered directly into the LV cavity via percutaneous injection under echocardiographic guidance. This method is much less traumatic than repeated thoracotomies, results in much higher survival rates, and enables CMCs to be delivered close to the coronary arteries (Guo et al., 2017; Nong et al., 2020). Using this method, we have previously documented that a significant number of cells are retained in the heart at 1 day after injection, comparable to or greater than that seen after intramyocardial and intracoronary injection (Guo et al., 2017).

The robustness of our conclusions is supported by a number of considerations. In almost all rodent studies, LV function is assessed only with load-dependent measurements. However, there are distinct advantages in using load-independent parameters. Unlike EF or fractional shortening, load-independent parameters are not affected by uncontrollable variables such as venous return, ventricular dimensions, aortic pressure, LV compliance, peripheral resistance, etc., which vary continuously and unpredictably in intact animals (Schertel, 1998; Lindsey et al., 2018). Consequently, load-independent parameters are more reliable and meaningful. Among these, particular importance should be assigned to end-systolic elastance (Ees), which is often regarded as the gold-standard for assessing cardiac contractility (Schertel, 1998; Lindsey et al., 2018). Therefore, in this study the measurements of Ees could be viewed as the most important indicator of the therapeutic actions of transplanted cells. As shown in Figure 6G, in the 5% O_2_ CMCs group Ees was significantly greater not only compared with the vehicle group but also compared with the 21% O_2_ CMCs group. The superiority of 5% O_2_ CMCs is further corroborated by the structural results. As shown in Figures 7, 8 and Table 2, structural improvement, i.e., a reduction in scar size, LV hypertrophy, and collagen content and an increase in viable myocardium, were observed only in hearts treated with 5% O_2_ CMCs. Taken together, these diverse and independent results support the conclusion that 5% O_2_ CMCs were more efficacious than 21% O_2_ CMCs.

The mechanism(s) responsible for the superior therapeutic efficacy of 5% O_2_ CMCs vs. 21% O_2_ CMCs remain to be elucidated. We have previously demonstrated that CMCs do not engraft in the heart and do not differentiate into new cardiomyocytes (Guo et al., 2017; Wysoczynski et al., 2017) and thus (similar to other cell types) (Tokita et al., 2016) act via paracrine mechanisms. The exact nature of these paracrine actions has not yet been ascertained for any mesenchymal cell type used heretofore (Banerjee et al., 2018). The increased resistance of 5% O_2_ CMCs to severe hypoxia and their much higher proliferation rate *in vitro* as well as their enhanced telomerase activity and elongated telomere length (Bolli et al., 2021) imply that, after transplantation *in vivo*, 5% O_2_ CMCs will be more likely to survive the harsh environment of the infarcted heart and will continue to multiply at a higher rate, which should result in greater cell numbers and thus greater therapeutic effects. Our finding that treatment with 5% O_2_ CMCs was associated with an increase in viable myocardium (Figure 7) suggests possible antiapoptotic actions and/or increase proliferation of endogenous myocytes. Our finding of decreased collagen content in both the risk and non-infarcted regions of hearts treated with 5% O_2_ CMCs (Figure 8) offers another possible explanation, since increased collagen deposition resulting in fibrosis impairs contractile function. Further studies will be necessary to test these hypotheses and determine the mechanisms that account for the superiority of 5% O_2_ CMCs. This uncertainty regarding mechanism(s) of action is common to essentially all cell types that have been tested for the treatment of heart failure (Wysoczynski et al., 2018).

## Conclusion

In conclusion, this study shows for the first time that, compared with the commonly used atmospheric oxygen tension (21%), the use of physiologic oxygen tension (5%) to culture CMCs markedly increases their therapeutic efficacy in a murine model of chronic ischemic cardiomyopathy. These results are important not only because they enhance the therapeutic potential of CMCs but also because culture at 5% O_2_ enables greater numbers of cells to be produced in a shorter time, thereby reducing costs and effort and limiting cell senescence (Bolli et al., 2021). Due to the similarity between CMCs and other stem/progenitor cells, it is likely to these results may apply to other types of cells being studied in experimental or clinical trials but still cultured at atmospheric O_2_ tension. Thus, the present study has potentially vast implications because it supports a paradigm shift in the field of cell therapy. The standard method to culture most stem/progenitor cells, including CMCs, is to use 21% O_2_ tension rather than the physiologic oxygen tension in the tissue. Our results suggest this method needs to be changed and that for CMCs, and probably for other types of stem/progenitor cells as well, physiologic oxygen tension needs to be used to maximize therapeutic efficacy, both in preclinical and clinical studies.

## Data Availability Statement

The original contributions presented in the study are included in the article, further inquiries can be directed to the corresponding author.

## Ethics Statement

The animal study was reviewed and approved by Institutional Animal Care and Use Committee, University of Louisville.

## Author Contributions

RB did cell culture, acquired cell images, and conducted echocardiographic studies and analyses as well as histological procedure. AA and SH performed histological sectioning, staining, and image analyses. CD did cell culture partially for cell transplantation. AT, YN, and YG performed the surgical procedures, echocardiography-guided intraventricular cell transplantation, echocardiographic and hemodynamic studies, and analyses. QL partially performed experiments, designed all the studies, analyzed and interpreted the data, prepared figures, and wrote and finalized the manuscript. All authors have reviewed and approved the final version of the manuscript and meet the International Committee for Medical Journal Editors (ICMJE) authorship criteria.

## Conflict of Interest

The authors declare that the research was conducted in the absence of any commercial or financial relationships that could be construed as a potential conflict of interest.
